# Low Prevalence of Enzootic Equine Influenza Virus among Horses in Mongolia

**DOI:** 10.3390/pathogens6040061

**Published:** 2017-11-30

**Authors:** Alexandra Sack, Ulziimaa Daramragchaa, Maitsetseg Chuluunbaatar, Battsetseg Gonchigoo, Boldbaatar Bazartseren, Nyamdorj Tsogbadrakh, Gregory C. Gray

**Affiliations:** 1 Institute of Veterinary Medicine, Mongolian University of Life Sciences, Ulaanbaatar 17024, Mongolia; ulzima_d@yahoo.com (U.D.); ch.maitsetseg@gmail.com (M.C.); gotsetseg@mail.mn (B.G.); boldoomglvet@yahoo.com (B.B.); 2 Division of Infectious Diseases, and Global Health Institute, Duke University, Durham, NC 27708, USA; gregory.gray@duke.edu; 3 National Center for Zoonotic Diseases, Ulaanbaatar 18131, Mongolia; tsogoo_0210@yahoo.com; 4 Global Health Research Center, Duke-Kunshan University, Kunshan 215316, China; 5 Program in Emerging Infectious Diseases, Duke-NUS Medical School, Singapur 169857, Singapore

**Keywords:** Bactrian camels, equine influenza, Mongolia, pastoralism

## Abstract

Horses are critically important for Mongolian herders’ livelihoods, providing transportation and food products, and playing important cultural roles. Equine influenza virus (EIV) epizootics have been frequent among Mongolia’s horses, with five occurring since 1970. We sought to estimate the prevalence for EIV infection among horses and Bactrian camels with influenza-like illness between national epizootics. In 2016–2017, active surveillance for EIV was periodically performed in four aimags (provinces). Nasal swabs were collected from 680 horses and 131 camels. Seven of the horse swabs were “positive” for qRT-PCR evidence of influenza A (Ct value ≤ 38). Two more were “suspect positive” (Ct value > 38 and ≤ 40). These nine specimens were collected from four aimags. None of the camel specimens had molecular evidence of infection. Despite serial blind passage in Madin-Darby Canine Kidney cells (MDCK) cells, none of the nine horse specimens yielded an influenza A virus. None of the 131 herder households surveyed had recently vaccinated their horses against EIV. It seems likely that sporadic EIV is enzootic in multiple Mongolian aimags. This finding, the infrequent use of EIV vaccination, periodic prevalence of highly pathogenic avian influenza, and the mixing of domestic and wild equid herds suggest that Mongolia may be a hot spot for novel EIV emergence.

## 1. Introduction

Mongolia’s relationship with domestic horses (*Equus ferus caballus*) is unique, as horses play important roles in transportation, cultural events, and as a food source for many of Mongolia’s rural people. With an estimated 3.3 million horses, there are likely more horses today in Mongolia than people [1,2]. In one survey in Tov aimag (province), 78.5% of people reported eating horse meat [3]. Airag (fermented horse milk) and various other milk products are central to Mongolia’s culture [4]. Alongside domestic horses, Mongolia is also home to Mongolian wild asses (*Equus hemionus hemionus*), and the majority of the remaining wild Takhi or Przewalski’s horses (*Equus ferus przewalskii*) [5,6].

This large population and critical economic role of horses as well as the daily interactions during most of the year between humans and horses in Mongolia results in increased risk of infectious disease transmission. One disease with the potential to cause severe epidemics in equids as well as to be transmitted to other species is equine influenza [7]. Equine influenza viruses (EIVs) are highly infectious among equids and sporadically detected worldwide [8]. EIV can be spread among horses by direct contact, through fomites, and by aerosol transmission [8,9,10]. Epizootics have been associated with the movement of horses for horse racing events. 

EIV has the potential to be economically debilitating even when the horse mortality is low. Horses usually recover with rest within two weeks of sign onset, but the loss of productivity can be prolonged. Secondary bacterial pneumonia may develop which can be fatal, particularly in young horses and foals [11,12]. Even without mortality, the required two weeks to a few months of rest may cause horse owners economic hardship. Of importance to conservation efforts, Takhi can also be infected by EIV. During a 2007 EIV outbreak in China, a Takhi herd had 100% morbidity and 5% mortality due to EIV infection [6]. 

Equine influenza is caused by influenza A virus, from the *Orthomyxoviridae* family. Two subtypes, H7N7 and H3N8, historically caused infections in horses. However, H7N7 has not been isolated in horses since the late 1970s [13]. EIV H3N8 is divided into European and American strains, with the American strains being further divided into Florida, Kentucky, and South America. Florida is further divided into two antigenically dissimilar clades [8]. Recent EIV outbreaks in China, Mongolia, and Kazakhstan were caused by clade 2 H3N8 EIV [14]. 

Historically in Mongolia, three outbreaks with high mortality rates have been recorded from the 1970s–1990s. These records estimate a 20–30% death rate for all three outbreaks. Vaccination was implemented starting with an outbreak in 2007–2008. Vaccination during the 2007–2008 outbreak was credited with a lower (5%) mortality. The most recent reported outbreak in 2011–2012 occurred in all 21 aimags [15]. The EIV vaccine used during the most recent outbreaks was quadrivalent: A/equine/Miami/63 (H3N8), A/equine/Mongolia/93 (H3N8), A/equine/Mongolia/83 (H3N8), and A/equine/Prague/56 (H7N7). Bactrian camel (*Camelus* bactrianus) in Mongolia also tested positive for an influenza virus of equine origin in 2012–2013 [16]. In this study, we sought to examine Mongolian horses and camels with signs of influenza-like illness (ILI) for evidence of EIV such that we might better understand EIV transmission between national epizootics. 

## 2. Results

### 2.1. Naadam Festival

Seventy horses with signs of ILI were tested during the Naadam festival from July 10–12, 2016 during six races. All horses had nasal discharge as their primary sign of ILI. Three samples were “positive” and two were “suspect positive” for influenza A virus by qRT-PCR (Table 1). Four out of five were typed as H3 influenza. No influenza A viruses were grown despite four passages in MDCK cells. The five horses were all from the youngest two age groups and their representing owners lived in three aimags: Uvs, Zavkhan, and Tov. 

### 2.2. Active Surveillance in Rural Mongolia

Six hundred and ten horses with ILI were swabbed and tested between January 2016 and March 2017. Between January–February and April 2016, 299 horses with ILI were tested from two aimags, Arkhangai (*n* = 40) and Uvurkhangai (*n* = 259). None were positive for influenza. During October–November 2016, 189 horses with ILI were tested from three aimags: Arkhangai (*n* = 79), Uvurkhangai (*n* = 66), and Umnugobi (*n* = 44). All six influenza A-positive horses were from Uvurkhangai aimag and from two soums (Khairkhandulaan and Taragt), with two families (three horses) in the first soum and one family (one horse) in the second soum (Table 2). No influenza A viruses were grown, despite four passages in MDCK cells. During February–March 2017, 122 horses were tested with ILI in three aimags: Arkhangai (*n* = 75), Uvurkhangai (*n* = 39), and Umnugobi (*n* = 8). None of the horses from 2017 were positive for EIV. In November 2016, 24 camels were tested and in February 2017, 107 camels were tested for influenza in Umnugobi. Camels ranged in age from less than a year old to 21 years old. None of the 131 camels tested positive for influenza.

**Figure 1:**
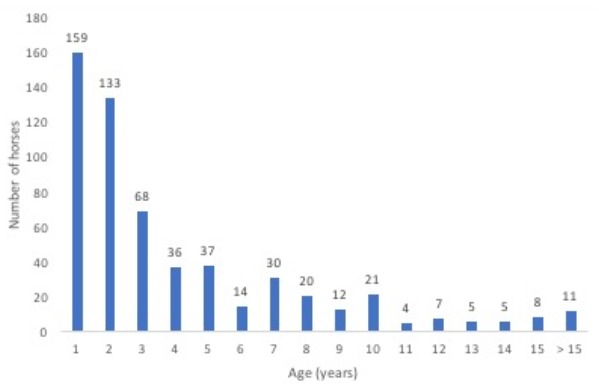
The age in years of sampled horses with influenza-like illness (*n* = 570).

In total, 680 horses with ILI were tested for EIV. Age was recorded from 570 of 610 horses, excluding the 70 horses from Naadam. Horses ranged in age from less than a year old to 31 years old. More than 50% of horses sampled with ILI were 2 years old or younger (Figure 1). Of the 159 horses that were 1 year old or less, 93.1% were tested in the fall.

Of the 311 horses with individually recorded signs between October 2016 and March 2017, the most common signs were ocular discharge (92.9%), nasal discharge (85.5%), and cough (17.4%, Table 3). Horses positive for EIV all had ocular discharge (four horses). Most also had nasal discharge (thre horses, 75%) and one (25%) presented with a cough (Table 2).

### 2.3. EIV and EIV Vaccine Awareness and Willingness to Pay

Overall, 112 of the 131 survey participants (85.5%) had heard about equine influenza virus (EIV). However, only 29 households (22.1%) reported knowing that there is a vaccine for EIV. In Arkhangai, only five households (9.8%) had heard about an EIV vaccine while only four households (13.3%) in Umnugobi knew of the vaccine. However, 40.8% (20) of participants in Uvurkhangai reported knowledge about an EIV vaccine. Of these 29 participants, nine (6.9%) had vaccinated their horses in the past, but none had vaccinated their horses in the previous year of this study. Seven of the nine households who had vaccinated for EIV in the past were in Uvurkhangai aimag, with one each in Arkhangai and Umnugobi. There was a significant difference between household aimag and a participant’s awareness of an EIV vaccine (χ^2^ = 15.998, *p* < 0.001). 

Of the 131 surveyed households, 126 (96.25%) reported that they would be willing to give an EIV vaccine to their horses if it was free and 125 (95.4%) reported they were willing to pay for a vaccine. When asked to cite a fair price, 128 households answered and the average was 179 Mongolian tugriks (MNT) ($0.07) per horse, with the most common answer being 100 MNT ($0.04). Only 10 households (7.8%) were not willing to pay at least 100 MNT per horse. There was a significant difference between what households in each aimag were willing to pay for an EIV horse vaccine (*p* = 0.008). Uvurkhangai was willing to pay an average of 239 MNT ($0.10), which was significantly more than both Arkhangai’s average of 153 MNT ($0.06; *p* = 0.033) and Umnugobi’s average of 123 MNT ($0.05; *p* = 0.001).

## 3. Discussion

Surveillance between outbreaks has been limited. A serologic study in 2007 before the EIV outbreak that year found that 8.5% of horses were serologically positive for H3N8 in Selenge, a northern aimag [17]. Our current study adds to existing knowledge regarding EIV prevalence in Mongolia between outbreaks. During this study, nine out of 680 (1.3%) of horses with ILI were positive for influenza A, and eight out of nine were typed as a H3 influenza. Two of these were suspect positives. If these horses were actively shedding virus (which we did not demonstrate), they indicate a continued transmission of EIV in Mongolia. 

### 3.1. National Horse Races at Naadam

Specifically, the three positive horses and two suspect positive horses from the national Naadam races demonstrate both an opportunity to prevent and a risk for future EIV outbreaks. These five horses were in the 2-year-old and yearling races. Worldwide shows and races are known to be associated with EIV outbreaks [8]. The horse races during the national Naadam festival were followed in 2011 with a national outbreak of EIV [15]. Even with this finding, we are not aware of any surveillance that has been done at Naadam for EIV before or after the races. The presence of EIV positive horses at the Naadam races shows that there is risk for another national outbreak, as naïve horses may be exposed to EIV and then travel back home. Naïve horses may shed large amounts of virus by coughing [18], and travel is known to stress horses and predispose them to illness [19]. It seems possible that the Nadam races may enhance the risk of novel EIV virus generation through the mixing of horses from different regions which might carry different EIV strains. 

Young horses are at an increased risk of EIV. All but one of the horses we found to be positive were in the yearling race (Table 1). Horses under the age of five are known to be more susceptible, and younger horses are more at risk in developing a secondary bacterial pneumonia [20]. This is even more of a risk for race horses, as long races are exhausting to many horses. Overworked horses are more susceptible to death from EIV or from a secondary bacterial infection [11]. Also, due to the sampling being at the start of the races, potentially more horses were positive, but their coughing or other signs were missed in the brief window of observation. There was one sample from the Naadam races that tested positive for Influenza A and not for H3. This is believed to be because the qRT-PCR for H3 was conducted more than six months later than the qRT-PCR for influenza A, and sample degradation may have occurred. 

### 3.2. Geographic Distribution

The five influenza-positive horses from the national Naadam horse races represented three different aimags. Three of the horses were registered to Ulaanbaatar, which was the owner’s address and did not necessarily indicate that the horses were from Tov aimag. It is possible then that even more than three aimags were represented. Outside of the national horse races, horses with influenza were found in Uvurkhangai, one of the three aimags in which active surveillance occurred (Table 2). All aimags, including the samples from Naadam, with EIV-positive horses in this study are found in the northern and western parts of Mongolia. The single aimag found to have horses positive for EIV serologically in 2007 is adjacent to the aimags found to have EIV-positive horses in this study [17]. However, no active surveillance was conducted in the eastern part of the country, so more surveillance would be necessary to determine the extent of EIV prevalence throughout Mongolia’s 29 aimags. 

### 3.3 South Gobi and Camels

No camels were found to be positive for EIV during this study. It is still unknown if the single Bactrian camel with EIV found in Mongolia was an aberrant event or part of the normal transmissions of EIV [16]. We also were surprised that no horses from Umnugobi aimag were positive for EIV. Umnugobi was chosen for this study as it is the aimag with the most camels in Mongolia [1]. 

### 3.4. Age and Signs of Horses with ILI and EIV

Of the horses with ILI and a recorded age, more than 50% were less than 2 years old. Ninety percent of horses that were 1 year old or younger were tested in the fall, when mares were still being milked [4]. In most cases, foals are tied in a row, so the mares will not wander away from the herders’ gers. This clustering of mares and foals may increase the risk of EIV transmission. Of the horses with influenza, all were 3 years old or younger, and seven out of nine (77.8%) were 1 year old or younger. All EIV-positive horses being less than 5 years old is consistent with the last EIV outbreak in Mongolia occurring in 2011, as at that time, extensive EIV vaccination was also employed [15]. The young age of EIV-positive horses combined with the low level of vaccination suggests that low levels of EIV infection may occur among younger horses, resulting in herd immunity. However, the historical record of EIV epizootics in Mongolia suggests that this herd immunity may not be not sufficient to prevent periodic national epizootics [15]. 

In this study, 321 horses with ILI had individually recorded signs. The most prevalent signs were nasal (92.9%) and ocular discharge (85.5%). Coughing (17.4%) was the third most prevalent sign. Ocular and nasal discharge were the easiest signs to identify among free range horses. Especially during winter when horses are often away from the gers, many herders did not see their horses daily or even weekly. Similarly, the most prevalent signs for horses that tested positive for EIV were ocular discharge (all four horses, 100%), followed by nasal discharge (three horses, 75%) and then cough (one horse, 25%).

Cough, nasal discharge, and pyrexia are the most commonly seen signs with EIV [18]. Which sign is most commonly seen may depend on multiple factors. One study found that all horse had a fever but often for a very short period of time. Nasal discharge was the most commonly noted sign with over 90% showing nasal discharge, and only over 50% coughing [21]. In another study, over 90% had a fever, nasal discharge, and cough. However, these three signs rarely occurred simultaneously [22]. These differences in signs are unlikely to be due to strain differences, as both studies were undertaken in Australia during the same 2007 EIV outbreak. In our study, no influenza-positive horses showed both cough and nasal discharge. Temperature was not measured because of safety concerns. Horses with these three signs should be considered highly suspicious for EIV, but even horses with two signs should be treated as suspect. This is especially true in Mongolia, as some signs may be easier to overlook when horses are minimally managed. 

### 3.5. Current Vaccination Status

In the three aimags that were sampled during active surveillance, no one had vaccinated their horses for EIV in the past year, and only nine (6.9%) reported ever vaccinating their horses for EIV. Only 29 households (22.1%) reported knowing that there is a vaccine for EIV, even though over 85% knew about EIV. There was a significant difference between aimags suggesting that investigation into the information source in Uvurkhangai could assist communications in the other aimags. However, over 95% of households reported that they would be willing to administer an EIV vaccine for their horses if it was free, and 115 households were willing to pay 100 MNT ($0.04). There is a desire to protect horses from EIV, but a gap exists between that desire and the current knowledge and practice.

### 3.6. Potential for Zoonotic Transmission

All nine influenza A-positive horses were tested in the fall and summer of 2016 (Table 1 and Table 2). Summer and fall are the seasons when mares are milked for airag production. Mares are milked five to seven times a day from late June until late summer/fall, depending on the geographic region [4]. As foals are also handled at this time, this potentially exposes the milk and herders to any EIV the foal may also be carrying. This increases the risk of an outbreak, as EIV is also spread on people’s clothing and via fomites [8]. There is also the serious concern of zoonotic transmission of existing or potentially new EIV strains. A recent review of the evidence of the zoonotic potential of EIV, specifically H3N8, found evidence in support of occasional zoonotic transmission of EIV. The same paper observed that horses have been infected with other strains of influenza, including H1N8, H5N1, H7N1, and H9N2 [23]. This increases the risk for the emergence of novel influenza viruses.

Many historical human influenza outbreaks had been preceded or were less commonly followed by equine outbreaks. One review of the historical records in Europe from 1688 to 1888 found 56 years with documented outbreaks of human or equine influenza. Out of the 56 outbreaks, both horses and humans were affected in 21 years, humans only in 25 years, and horses only in 10 years. Some of the largest EIV outbreaks with smaller accompanying human influenza outbreaks occurred in 1727, 1750, 1760, and 1872. These are historical records of ILI, as confirmatory diagnostic tests did not exist [24]. Human and equine EIV outbreaks occurred in the past, and a novel EIV or reemergence of H7N7 could allow them to return [23]. 

### 3.7. Study Limitations

This study has a number of limitations. The periodic sampling and focus upon only four of 29 aimags limits our ability to apply our findings to all of Mongolia. Cold-chain problems and specimen transportation issues likely decreased our molecular assay and virus yield in cell culture yields. Nasal swabs were chosen over nasopharyngeal swabs as the latter method often required sedation and was not feasible with the infrastructure and resources available. Our inability to grow live influenza A virus from the swab specimens that were molecularly positive could have a number of explanations including low or no viability.

### 3.8. Future Directions and Conclusions 

EIV surveillance is important in designing a strategy for reducing morbidity and mortality, as well as ameliorating any potential novel EIV emergence and zoonotic transmission. In Mongolia, EIV-positive horses were found in 2016 at the national Naadam horse race as well as in the countryside in the fall of 2016. Young, EIV-positive horses at Naadam are at risk for mortality due to stress from traveling and racing [11,20]. There is also a risk for another national outbreak or the emergence of novel EIV, as horses from a large geographic area intermix. Vaccination is thus suggested as a method to reduce the clinical illness from EIV, as well as reduce viral shedding [8]. More than 95% of herder families were willing to pay for a vaccine, but none had vaccinated their horses for EIV in the last year.

Outside of race horses, EIV still has the potential to be devastating to Mongolian herder families. In this study, four influenza-positive horses were found in Uvurkhangai during the fall of 2016. With mares being milked for airag, EIV in the summer and fall has the potential to affect families economically. 

This study has thus shown that EIV is endemic in Mongolia between large-scale outbreaks. Further surveillance is necessary to see if these seasonal and geographical patterns extend outside of 2016, and to help best plan for EIV prevention in race horses and other horses in Mongolia. This endemic status combined with most equines receiving no routine vaccination may lead Mongolia to serve as an enzootic geographical area for novel EIV emergence, as well as potential zoonotic transmission.

## 4. Methods

### 4.1. Sample Collection

**Figure 2:**
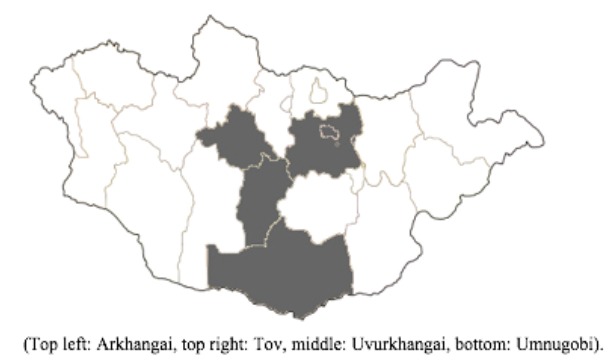
The four aimag (province) study sites.

Sample collection for our surveillance was periodically conducted in four aimags: Tov, Uvurkhangai, Arkhangai, and Umnugobi (Figure 2). These four aimags were chosen for their high density of livestock, geographic diversity, and for the national Naadam horse races which occur each summer in Tov. Local veterinarians in the aimags and soums (towns) assisted with locating and contacting the participating herder families, as well as assisted in collecting nasal swab samples.

This project received ethical approval from Duke University’s Institutional Animal Care and Use Committee (IACUC), as well as full approval from the Mongolian Monitoring Committee of Medical Ethics—Ministry of Health (for the herder family survey). 

Nasal swabs were collected from horses with signs of ILI. ILI was defined as two or more of the following signs: nasal discharge, ocular discharge, cough, fatigue, enlarged lymph nodes, hyporexia, and/or abscesses along the upper respiratory tract. In Umnugobi aimag, nasal swabs were also taken from Bactrian camels with nasal and ocular discharge. Training was conducted in Ulaanbaatar and later in the rural aimag among rural veterinary and public health staff. Depending upon the aimag training, surveillance began during the period of January to April 2016 and continued sporadically through March 2017. When the Ulaanbaatar-based team periodically visited the rural sites, herder families would gather the free-ranging horses and camels for screening. Restraint was normally achieved by halters, ropes, or hand restraint. Samples were not taken if it was deemed unsafe for either the human, the horse, or the camel. In all cases, two nasal swabs were collected from each horse or camel. Location and age was recorded for each horse or camel swabbed. A maximum of 10 horses and/or camels was sampled per herder family.

During Naadam (July 2016), as guided by Nadam equine veterinary staff, only one of the above signs was used to trigger nasal swab collections before races began. After horses completed their races, the team reverted to selecting horses with two or more signs of illness. Nadam sampling was conducted at Khui Doloon Khudag in Tov aimag. Horses were not sampled if the procedure was deemed unsafe or if the jockey could not stop the horse. 

After collection, swabs were placed into a 16 × 100-mm screwcap conical tube containing 3 mL of universal transport medium and transported in refrigerated boxes until frozen at −80 °C. Samples were aliquoted into two 1.5-mL tubes and placed in liquid nitrogen for transport. Long-term storage of samples was at −80 °C.

### 4.2. Surveys

The head of each herder household was asked to respond to a series of oral questions from a brief Mongolian language household survey. Data were analyzed using Microsoft Excel 2016 and Epi Info 7.1.5.2. Descriptive statistics were run to characterize data averages and ranges. Differences in willingness to pay were analyzed with a one-way analysis of variance and pairwise post hoc test, as the data was normally distributed. 

### 4.3. Nucleic Acid Extraction

Viral RNA was isolated from 140 µL taken from each swab sample. The samples were then processed using the Qiagen: QIAamp Viral RNA Mini Kit (Qiagen Inc., Valencia, CA, USA) following a mini-spin protocol. The extracted nucleic acids were eluted into 60 µL of elution buffer provided in the QIAamp Viral RNA Mini Kit. 

### 4.4. Influenza Virus Detection and Characterization 

A real-time PCR (qRT-PCR) procedure designed by the World Health Organization/U.S. Center for Disease Control (CDC, Atlanta, GA, USA) was used to screen respiratory specimens for any influenza A virus [25]. This procedure detected influenza A (all types) using a specific primer for the M gene. All primers were purchased from Integrated DNA Technologies (Coralville, ID, USA).

Thermocycling was performed using a BioRad real-time PCR detection system with 96-well format. All qRT-PCR runs had a template negative control and the corresponding primer set viral template positive control. Each extraction run included a mock extraction control to provide a second negative control to validate the extraction procedure and reagent integrity. Specimens that were positive by qRT-PCR for influenza A were further evaluated with the U.S. Center for Disease Control’s (CDC) qRT-PCR H3-specific assay. As per World Health Organization guidelines, “positive” assay results were defined as a qRT-PCR Ct value ≤ 38 and “suspect positive” results were defined as a Ct value between 38 and 40 [26]. These cutpoints are used by Mongolia’s National Influenza Center as guided by the U.S. CDC.

Virus isolation was attempted from the six RT-PCR positive samples using Madin-Darby canine kidney cells (MDCK) obtained from Nihon University in Tokyo, Japan. Cells were cultured in T-25 flasks with antibiotics (penicillin, streptomycin, fungizone) and fetal bovine serum (FBS) at 37 °C for 3–4 days. Once cells became confluent (80–90%), swab supernatants were filtered with a 450-nm pore sized filter, then 200 µL of filtered supernatant was inoculated into cells and incubated for 30 min at 37 °C for adsorption. After adsorption, 6 mL of complete media (D-MEM) containing 2 µg/mL of trypsin without calf serum was added to T-25 flasks. Cytopathogenic effect in cells was checked daily for 7 days each for four passages.

## Figures and Tables

**Table 1:** Influenza molecular assays results among horses with nasal discharge at the national Naadam horse races in 2016, by race.

Race	Number Tested	qRT-PCR Positives for Influenza A (%)	qRT-PCR Positives for H3
Daaga (1 years)	18	4 (22.2) *	4
Shudlen (2 years)	11	1 (9.1)	0
Hizaalan (3 years)	13	0 (0)	0
Soyolon (4 years)	6	0 (0)	0
Azraga (5 years)	10	0 (0)	0
Ih nas (older than 5)	12	0 (0)	0
Total	70	5 (7.1)	4

* Two samples were positive and two were suspect positive.

**Table 2:** Horses with signs of influenza-like illness that were positive for H3 influenza A by qRT-PCR.

Aimag	Date	Soum	Influenza A- and H3-Positive Horses Per ILI Horses in Herd	Age	Cough	Nasal Discharge	Ocular Discharge
Uvurkhangai	9 October 2016	Khairkhandulaan	1/6	3	Yes	No	Yes
Uvurkhangai	9 October 2016	Khairkhandulaan	2/2	1	No	Yes	Yes
Uvurkhangai	9 October 2016	Khairkhandulaan	2/2	1	No	Yes	Yes
Uvurkhangai	10 October 2016	Taragt	1/8	1	No	Yes	Yes

**Table 3:** Signs of horses sampled with influenza-like illness (ILI) between October 2016 and March 2017.

Signs	Number Tested (%)
Ocular discharge	289 (92.9)
Nasal discharge	266 (85.5)
Cough	54 (17.4)
Fatigue	5 (1.6)
Hyporexia	3 (1.0)
Enlarged submandibular lymph nodes	1 (0.3)
Total	311
